# Structures of the *Neisseria meningitides* methionine‐binding protein MetQ in substrate‐free form and bound to l‐ and d‐methionine isomers

**DOI:** 10.1002/pro.3694

**Published:** 2019-08-09

**Authors:** Phong T. Nguyen, Jeffrey Y. Lai, Jens T. Kaiser, Douglas C. Rees

**Affiliations:** ^1^ Division of Chemistry and Chemical Engineering California Institute of Technology Pasadena California; ^2^ Howard Hughes Medical Institute and Division of Chemistry and Chemical Engineering California Institute of Technology Pasadena California

**Keywords:** ABC transporters, d‐methionine, l‐methionine, MetNI methionine importer, periplasmic binding protein MetQ

## Abstract

The bacterial periplasmic methionine‐binding protein MetQ is involved in the import of methionine by the cognate MetNI methionine ATP binding cassette (ABC) transporter. The MetNIQ system is one of the few members of the ABC importer family that has been structurally characterized in multiple conformational states. Critical missing elements in the structural analysis of MetNIQ are the structure of the substrate‐free form of MetQ, and detailing how MetQ binds multiple methionine derivatives, including both l‐ and d‐methionine isomers. In this study, we report the structures of the *Neisseria meningitides* MetQ in substrate‐free form and in complexes with l‐methionine and with d‐methionine, along with the associated binding constants determined by isothermal titration calorimetry. Structures of the substrate‐free (N238A) and substrate‐bound *N*. *meningitides* MetQ are related by a “Venus‐fly trap” hinge‐type movement of the two domains accompanying methionine binding and dissociation. l‐ and d‐methionine bind to the same site on MetQ, and this study emphasizes the important role of asparagine 238 in ligand binding and affinity. A thermodynamic analysis demonstrates that ligand‐free MetQ associates with the ATP‐bound form of MetNI ∼40 times more tightly than does liganded MetQ, consistent with the necessity of dissociating methionine from MetQ for transport to occur.

## 1 INTRODUCTION

The MetNI methionine ATP binding cassette (ABC) transporter from *Escherichia coli* was originally identified by Kadner et al^1^, ^2^, ^3^, ^4^ as mediating the high‐affinity uptake of l‐methionine, while exhibiting broad specificity toward other methionine derivatives, including d‐methionine, at lower affinities. MetQ, the substrate‐binding protein (SBP) for MetNI,^5^, ^6^ is proposed to play a dual role in transport by either binding l‐methionine for delivery to the translocation pathway of MetNI, or by facilitating d‐methionine uptake through the ligand‐free form when complexed to the transporter.^7^

As for all SBPs (reviewed in References 8, 9, 10), the MetQ structure^11^, ^12^, ^13^, ^14^, ^15^ consists of two domains connected by a hinge region, with the substrate‐binding site formed at the interface between the domains. Structural studies of SBPs in their ligand‐bound and ligand‐free forms have revealed a hinge‐bending or “Venus‐flytrap” mechanism where two domains of SBPs adopt their open conformation with an accessible binding site in the absence of substrate; substrate binding stabilizes the closed conformation, where the two domains come together and sequester the substrate.^16^, ^17^ Other types of conformational changes are possible for SBPs; for example, instead of a hinge‐bending motion, the ligand‐free MetQ in the *E*. *coli* MetNIQ complex exhibits a distinct type of conformational change relative to the holo‐MetQ, corresponding to a twist around an axis perpendicular to the interface between domains.^7^

The structures of the distinct conformational states of MetQ, together with a determination of the thermodynamics of ligand binding, are essential for a detailed understanding of the role of this binding protein in methionine uptake by MetNI. While multiple structures of MetQ methionine‐binding proteins have been determined,^11^, ^12^, ^13^, ^14^, ^15^ they all exhibit the closed conformation with bound l‐methionine, since MetQ has a high affinity for l‐methionine and is always isolated in the liganded form. With the exception of MetQ found in the MetNIQ complex, the structure of substrate‐free MetQ has not been reported. To fill this gap in our characterization of the MetNIQ transport system, we report the structures of *Neisseria meningitides* MetQ in the substrate‐free state, and in complexes with d‐methionine and with l‐methionine, together with the relevant dissociation constants.

## 2 RESULTS AND DISCUSSION

**Figure 1:**
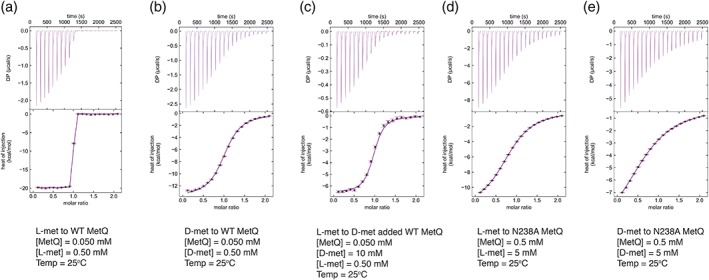
Isothermal titration calorimetry titrations of l‐methionine and d‐methionine binding to MetQ variants. ITC titrations of (a) the binding of l‐methionine to wild‐type MetQ; (b) the binding of d‐methionine to wild‐type MetQ; (c) displacement ITC titration of the binding of l‐methionine to wild‐type MetQ in the presence of d‐methionine; (d) the binding of l‐methionine to substrate‐free N238A *Neisseria meningitides* MetQ; (e) the binding of d‐methionine to N238A MetQ. The derived dissociation constants and enthalpies are presented in Table 1. Protein concentrations of MetQ ([MetQ]), l‐methionine ([l‐met]), and d‐methionine ([d‐met]) are shown in the figures. ITC, isothermal titration calorimetry

Since MetQ has a high affinity for l‐methionine, preparation of the apo‐MetQ requires cycles of unfolding and refolding to remove the bound ligand.^11^ As an alternative approach, mutation of Asn229 to Ala (N229A) in the binding pocket of the *E*. *coli* MetQ was found to substantially decrease the affinity toward methionine, thereby greatly facilitating preparation of the substrate‐free form of MetQ. Despite extensive efforts with both approaches, we could not crystallize the substrate‐free form of *E*. *coli* MetQ. While screening structurally characterized MetQs from the RCSB Protein Data Bank, we were able to prepare and crystallize a substrate‐free form of the *N*. *meningitides* MetQ (PDB 3IR1 14), containing the Asn to Ala mutation at residue 238 (N238A; corresponding to N229A of the *E*. *coli* MetQ) to disrupt substrate binding. As this asparagine residue interacts with both the α‐amino and α‐carboxyl groups of l‐methionine, mutation to alanine would be expected to significantly impair ligand binding. Indeed, isothermal titration calorimetry (ITC) studies of ligand binding to the wild type and N238A forms of *N*. *meningitides* MetQ quantitate the impact of this mutation on the binding of l‐methionine, with the dissociation constant (*K*_d_) changing by a factor of over 10^5^, from 0.2 nM to 78 μM; the binding of d‐methionine is also impaired, but to a lesser extent as the *K*_d_ changes from 3.5 to 240 μM (Figure 1 and Table 1).

**Table 1:** Summary of dissociation constants (*K*_d_) and Δ*H*s derived from ITC titrations

Proteins	Ligands	*K*_d_ (μM)	Δ*H* (kJ mol^−1^)	Incompetent fraction^a^
Wild‐type	l‐methionine	0.00020 [0.00017, 0.00034]^b^	−83 [−84, −81]	0.06 [0.049–0.067]^b^
Wild‐type	d‐methionine	3.5 [2.5, 4.6]	−55 [−57, −53]	0.06 [0.049–0.067]^b^
N238A	l‐methionine	78 [69, 89]	−42 [−50, −37]	0.04 [0, 0.067]^b^
N238A	d‐methionine	240 [190, 310]	−31 [−34, −29]	0.31 [0.28–0.34]^b^

Abbreviation: ITC, isothermal titration calorimetry.

a Incompetent fraction is the fraction of MetQ that is apparently not able to bind to titrant.

b About 68.3% confidence intervals determined by error‐surface projection^18^ are shown in square brackets.

**Figure 2:**
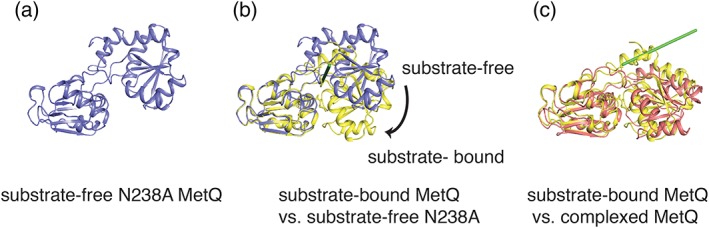
Conformational states of MetQ. (a) The structure of *Neisseria meningitides* MetQ in the substrate‐free (N238A) form depicted in a ribbons representation. (b) Superposition of one domain of the l‐methionine‐bound (tv_yellow) and N238A substrate‐free (slate) forms of MetQ, revealing a hinge‐type rotation axis (green axis, approximately parallel to the viewing direction) interconverting the two conformations. (c) The hinge movement is distinct from the twist‐type rotation axis (green axis, in the plane of view) interconverting the substrate‐bound form of MetQ and the substrate‐free conformation observed in the MetNIQ complex^7^

The crystal structure of the substrate‐free N238A MetQ was determined at 1.56 å resolution (Figure 2a, PDB 6CVA) and reveals an open conformation with an accessible substrate cavity. Superposition of one domain of the l‐methionine bound MetQ (PDB: 3IR1) on the corresponding domain of substrate‐free N238A MetQ reveals a 42° hinge‐type rotation that separates the two SBP domains and opens up the substrate‐binding cavity (Figure 2b). This “Venus‐fly trap” hinge movement contrasts with that observed for the relationship between liganded MetQ and substrate‐free MetQ in the MetNIQ complex, that are related by a 24° twist around an axis perpendicular to the interface between the two lobes.^7^ The root mean square deviation (rmsd) in Cα positions between the substrate‐free N238A and the l‐methionine bound form of *N*. *meningitidis* MetQ is 3.7 å, while the corresponding rmsd between the two substrate‐free forms of MetQ (N238A and the conformation of MetQ in complex with MetNI, PDB 6CVL) is 4.4 å.

**Figure 3:**
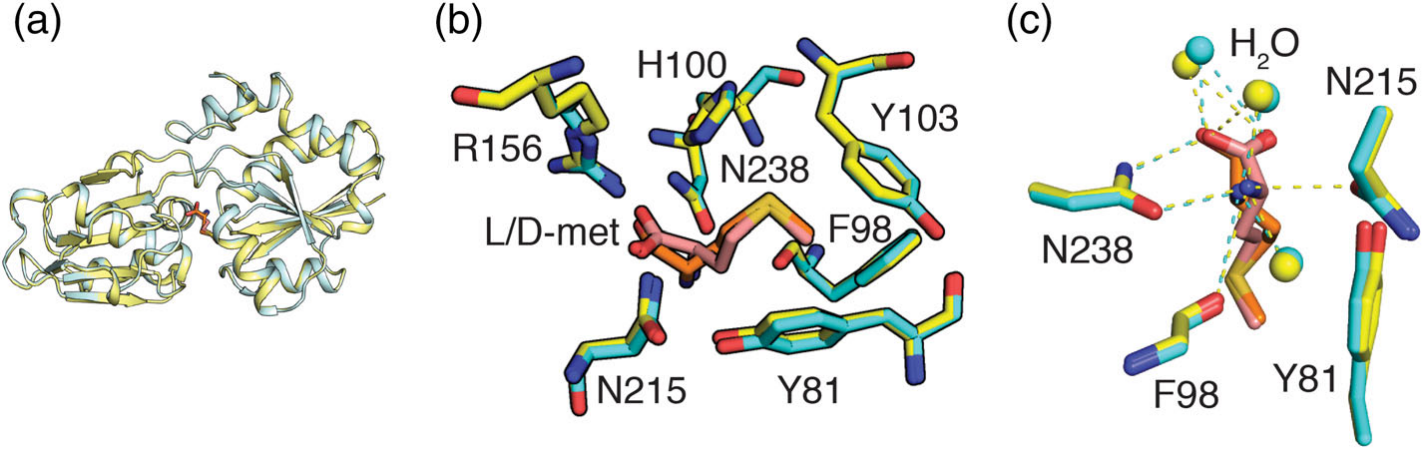
Binding of methionine isomers to *Neisseria meningitides* MetQ. (a) Superposition of MetQ with bound d‐methionine (cyan) and with bound l‐methionine (tv_yellow) depicted in a ribbons representation. (b) Superimposed binding sites for d‐methionine (orange) and l‐methionine (salmon) with surrounding residues shown in stick representation. (c) Hydrogen bonding interactions of the α‐amino and ‐carboxyl groups of the d‐methionine (cyan dashes) and l‐methionine (yellow dashes) to the neighboring residues of the d‐methionine (cyan sticks) and l‐methionine (yellow sticks) bound MetQ

MetQ can bind to other methionine derivatives, including d‐methionine, but preparing these forms has been complicated by the high affinity for l‐methionine. For example, a previous effort to crystallize d‐methionine‐bound MetQ was made by Yang et al^14^ by growing a methionine auxotroph *E*. *coli* B384 strain in the media containing 50 mg L^−1^ of d‐methionine. Due to the presence of amino acid racemases in *E*. *coli*, however, they obtained the l‐methionine bound structure.^14^ Hence, to crystallize the d‐methionine bound *N*. *meningitides* MetQ, we developed an alternative approach by unfolding/refolding MetQ during the MetQ purification to remove the endogenous bound l‐methionine. A sample of unfolded/refolded MetQ was then mixed with 10 mM d‐methionine prior to the crystallization. The crystal structure of the d‐methionine bound MetQ was solved at 1.68 å resolution with a clear density of d‐methionine in the binding cavity (Table 2, PDB 6DZX). Six molecules are present in the asymmetric unit of the d‐methionine bound MetQ crystal. As a control, we also determined the structure of the l‐methionine bound *N*. *meningitides* MetQ crystallized under similar conditions (Table 2, PDB 6OJA). Superposition of the l‐ and d‐methionine bound MetQ structures reveal their similar conformation, with rmsd ∼0.1 å (Figure 3a). While these structures are similar to the previously determined 3IR1 structure,^14^ rmsd ∼0.4 å, the space groups and packing interactions are distinct in these two studies.

Not surprisingly, l‐ and d‐methionine bind to the same site, interacting with the same set of conserved residues. This observation is consistent at all six MetQ molecules present in the asymmetric unit. The α‐amino and α‐carboxyl groups of both l‐ and d‐methionine interact with residues R156, N213, and N238 located on one domain of MetQ, while from the other lobe, residues Y81, F98, H100, and Y103 pack around the methionine thioether group (Figure 3b). These binding residues are highly conserved among different bacterial MetQ homologs.^11^, ^12^, ^13^, ^14^ The hydrogen bonds between the N238 side chain and the methionine α‐amino and α‐carboxyl groups appear to contribute significantly to the binding affinity, since mutation of this residue to alanine weakens the binding of l‐methionine by over 10^5^. Despite binding to the same site on MetQ, the detailed interactions for d‐ and l‐methionine differ as reflected in the ∼10^4^‐fold difference in *K*_d_ values. The origin of this difference in binding affinity is difficult to identify since the hydrogen‐bonding network involving the α‐amino and α‐carboxyl groups is largely unchanged between the two structures, including the positioning of water molecules (Figure 3c). Short contacts to the methionine CB group are observed in both structures, with ∼3.3 å distances observed to the Y98 CO in the l‐methionine structure, and to the Y81 OH groups in the d‐methionine structure. The side chains of l‐ and d‐methionine adopt distinct rotameric conformations for the N‐Cα‐Cβ‐Cγ; Cα‐Cβ‐Cγ‐Sδ; and Cβ‐Cγ‐Sδ‐Cε torsion angles, which are approximately −175°, −175°, and −70° for l‐methionine and +170°, −80°, and −60° for d‐methionine; the latter values correspond to −170°, +80°, and + 60°, respectively, for l‐methionine. Hence, while l‐ and d‐methionine exhibit distinct “ttm” and “tpp” rotamers, these rotamers are found in comparable abundances in proteins (∼6% each^19^), suggesting that the side chain torsion angle conformation does not contribute significantly to the differences in binding affinity (for reference, the most common methionine rotamer is “mmm” with a 20% frequency). Consequently, while there are differences in the details of the binding interactions between l‐ and d‐methionine to NmMetQ, a qualitative evaluation does not identify any obvious feature as dominating the differences in binding affinities for these two ligands.

The binding constants determined in this work provide an outline of the MetNIQ transport thermodynamics. MetQ has the highest affinity for the ATP‐bound form of MetNI, with dissociation constants to *E*. *coli* MetNI measured to be 1.1 μM and 27 nM for l‐methionine‐bound and substrate‐free N229A forms of *E*. *coli* MetQ, respectively.^11^ The sequence of binding events between MetQ and MetNI can be described by the equilibrium model in Equation (1), where *E*, *Q*_met_, and *Q* denote MetNI in the ATP‐bound form, methionine bound MetQ, and substrate‐free MetQ, respectively:

(1) $$\begin{array}{c} E+Q_{\text{met}}\overset{K_{1}}{⇌}{\mathrm{EQ}}_{\text{met}}\overset{K_{2}}{⇌}\mathrm{EQ}+\text{met}\overset{K_{3}}{⇌}E+Q+\text{met}\\ Q_{\text{met}}\overset{K_{4}}{⇌}Q+\text{met} \end{array}$$

*K*_1_ and *K*_3_ were previously determined for the *E*. *coli* MetNIQ system to be 1/1.1 μM = 9.1 × 10^5^ M^−1^ and 2.7 × 10^−8^ M, respectively,^11^ while *K*_4_ for l‐methionine is measured in this study as 2 × 10^−10^ M. The dissociation constant of the MetNIQ complex for l‐methionine, *K*_2_, may then be calculated as *K*_4_/(*K*_1_ *K*_3_) ∼10^−8^ M, so that the relative affinities of MetQ and MetNIQ for l‐methionine are:

(2) $$\frac{\left(Q_{\text{met}}\right)/\left(Q\right)}{\left({\mathrm{EQ}}_{\text{met}}\right)/\left(\mathrm{EQ}\right)}=\frac{1}{K_{2}K_{4}}=\frac{1}{K_{1}K_{3}}=41$$

The higher affinity of ATP‐bound MetNI for ligand‐free MetQ is not surprising given the high affinity of MetQ for l‐methionine (they co‐purify) and the necessity to dissociate the ligand from MetQ for transport to occur.

Three distinct conformational states have been characterized to date for MetQ: one liganded and two ligand‐free forms. The findings of a recent single molecule FRET study illustrating the conformational richness of SBPs, and the role of conformational dynamics in substrate transport,^20^ suggests that additional conformations of MetQ may be identified, perhaps for larger methionine derivatives with modified amino or carboxyl groups,^1^, ^2^, ^3^, ^4^ or in complexes with different states of the MetNI transporter. This study provides a foundation for addressing the coupling between the conformation of MetQ, the affinities of MetQ for MetNI and methionine derivatives that is at the heart of the specificity of ligand transport by the methionine transporter; an important next step is to define the kinetics of these interactions and deciphering how MgATP hydrolysis is coupled to ligand translocation.

## 3 MATERIALS AND METHODS

### 3.1 Cloning, expression, and purification

The *metQ* gene from *N*. *meningitides*, encoding for the mature MetQ without the signal sequence, was cloned into a modified pET21b (+) plasmid with N‐terminal deca‐histidine tag followed by an enterokinase‐cleavage site. The N238A mutation in MetQ was introduced by site‐directed mutagenesis (Stratagene). The cloned plasmids were expressed separately in *E*. *coli* BL21‐gold (DE3) cells (EMD) at room temperature in ZY media.^21^

Purification of N238A MetQ was performed by resuspending 10 g cell paste in 100 mL of lysis buffer containing 20 mM Tris–HCl pH 8, 100 mM NaCl, 10% glycerol, 5 mM β‐mercaptoethanol (βMe), 20 μg/mL DNAse I, 200 μg/mL lysozyme. Cell lysis was achieved by freezing and thawing for three cycles in liquid nitrogen and in a 42°C water bath, respectively. Clearing of the cell lysate was accomplished by centrifugation at 37,500*g*, 30 min, and 4°C. After the supernatant was collected, 70 mM imidazole pH 8.0 was added and the sample was then loaded onto a 5 mL Ni‐sepharose HP column (GE Healthcare) equilibrated with purification buffer containing 20 mM Tris–HCl pH 7.5, 100 mM NaCl, 70 mM midazole. After sample loading, 12 column volumes of the same buffer were pumped through the column to wash nonspecifically bound proteins off the Ni beads. Two column volumes of the elution buffer containing 20 mM Tris–HCl pH 7.5, 100 mM NaCl, 300 mM imidazole were used to elute protein off the Ni‐column. The eluate was then subjected to size‐exclusion chromatography. The eluted peaks were pooled, incubated with enterokinase (NEB) to cleave the His‐tag, followed by passage through a hand‐packed Ni‐column to remove MetQ retaining the His‐tag. The His‐tag cleaved MetQ was then concentrated to 20 mg/mL and flash‐frozen in aliquots of 300 μL per tube.

### 3.2 Removal of substrate from wild‐type *N. meningitides* MetQ

To remove the bound l‐methionine from wild‐type MetQ, we used the previously described unfolding/refolding procedure^11^ with minor modifications. In brief, following binding to a Ni‐NTA column, the sample was washed with 12 column volumes of denaturing buffer containing 6 M guanidine‐HCl, 20 mM HEPES pH 7.5, 100 mM NaCl, at a rate of 1.5 mL/min, to remove bound l‐methionine. To refold MetQ, the guanidine‐HCl was slowly removed by flowing renaturation buffer (20 mM HEPES pH 7.5, 100 mM NaCl) over the column at 1 mL/min for 90 min. The column was further washed with 10 column volumes of renaturation buffer. Finally, MetQ was eluted in 20 mM HEPES pH 7.5, 100 mM NaCl, and 300 mM imidazole, and passed through a size‐exclusion column (Superdex 200 16/60, GE Healthcare) equilibrated in 20 mM HEPES pH 7.5 and 100 mM NaCl. The monodisperse peak was collected and concentrated to 20 mg/mL using an Amicon 10‐kD MWCO concentrator (Millipore). The His‐tag cleaved MetQ was then concentrated again to 20 mg/mL and flash‐frozen in aliquots of 300 μL per tube.

### 3.3 Selenomethionine‐substituted proteins

To prepare selenomethionine‐substituted N238A MetQ protein, the cloned plasmid was transformed in *E*. *coli* auxotroph B834 (DE3) cells (EMD), which was then grown in PASM autoinduction media,^21^ containing 125 μg/mL selenomethionine for 3–5 days at room temperature.

### 3.4 Crystallization of the MetQ variants

Crystallization conditions for different MetQ variants were screened by vapor diffusion in hanging drops at 20°C at a protein concentration of 20 mg/mL, using the JCSG‐plus screen (Molecular Dimension). The ligand‐free N238A MetQ from *N*. *meningitidis* was crystallized from a condition containing 0.2 M MgCl_2_, 0.1 M Bis–Tris pH 5.5, 25% PEG3350. Crystals, fully grown after 5–7 days, were cryo‐protected by increasing the PEG concentration to 35% in increments of 5% and flash‐frozen for data collection. Selenomethionine‐substituted N238A MetQ crystals often diffracted better than the native ones.

To crystallize d‐methionine bound and l‐methionine bound MetQ, 10 mM d‐ or l‐methionine were added to a sample of ligand‐free (unfolded/refolded) MetQ at a concentration of 20 mg/mL. The samples were incubated for 30 min prior to crystallization. Initial hits of d‐ or l‐methionine bound MetQ crystals were grown from a condition containing 2.4 M (NH_4_)_2_SO_4_, 0.1 M sodium acetate pH 4.6. Crystal growth was optimized by preparing crystallization reservoir in D_2_O instead of H_2_O. The optimized reservoir contains 2.3 M (NH_4_)_2_SO_4_, 0.1 M sodium acetate pH 5. Crystals, fully grown after 5–7 days, were cryo‐protected in the same crystallization reservoir supplemented with 25% glycerol in increments of 5% and flash‐frozen for data collection.

### 3.5 Data collection and structure determination

All X‐ray diffraction data sets were collected at the Stanford Synchrotron Radiation Laboratory beamline 12–2, equipped with a PILATUS 6 M PAD detector. Diffraction images were processed and scaled with XDS,^22^ with data processing statistics in Table 2. To determine the structure of ligand‐free N238A MetQ, initial phases were obtained by experimental phasing (AutoSol, Phenix)^23^ using single anomalous dispersion data from a 1.56 å resolution selenomethionine derivative crystal. Model building was done with the Autobuild function (phenix.autobuild, Phenix) followed by manually building several missing residues in Coot 0.8.9 24 and refining using phenix.refine (Phenix). The final model of N238 MetQ fit the density well with *R*_work_/*R*_free_ = 0.18/0.22. Final refinement statistics are in Table 2.

**Table 2:** Data collection and crystal structure refinement statistics

Crystal	d‐methionine bound Nm MetQ	l‐methionine bound Nm MetQ	Substrate‐free NmMetQ
Wavelength (å)	0.9794	0.9792	0.9794
Resolution range (å)	34.31–1.678 (1.738–1.678)	34.2–1.547 (1.602–1.547)	34.22–1.559 (1.615–1.559)
Space group	P 1	P 1	P 2_1_ 2_1_ 2
Unit cell (a b c (å), α β γ (°))	79.66, 87.93, 91.64, 114.83, 104.12, 105.39	79.58, 87.65, 91.63, 114.70, 104.41, 105.24	52.56, 89.66, 45.088
Total reflections	3,103,914 (143,999)	1,954,462 (86,262)	203,203 (7,841)
Unique reflections	219,472 (21,547)	277,550 (13,387)	30,754 (2,741)
Multiplicity	14.1 (13.9)	7.0 (6.4)	6.5 (5.4)
Completeness (%)	96.04 (91.50)	95.95 (93.74)	98.77 (89.75)
Mean *I*/sigma(*I*)	10.5 (0.7)	18.8 (1.7)	10.4 (2.4)
Wilson B‐factor (å^2^)	27.47	23.04	17.16
R‐merge	0.132 (4.454)	0.042 (0.906)	0.167 (3.329)
R‐meas	0.137 (4.635)	0.049 (1.086)	0.181 (3.692)
R‐pim	0.036 (1.249)	0.018 (0.422)	0.071 (1.551)
CC1/2	0.999 (0.294)	1 (0.723)	0.995 (0.182)
Reflections used in refinement	218,427 (20,806)	27,7,479 (27,097)	30,752 (2741)
Reflections used for R‐free	11,054 (954)	13,991 (1,396)	2,000 (179)
R‐work	0.177 (0.355)	0.162 (0.263)	0.188 (0.258)
R‐free	0.201 (0.366)	0.180 (0.296)	0.220 (0.291)
Number of non‐hydrogen atoms	12,841	13,172	2,143
Macromolecules	11,454	11,448	1,895
Solvent	1,387	1,724	248
Protein residues	1,451	1,452	241
RMS bonds (å)	0.010	0.005	0.003
RMS angles (°)	1.11	0.92	0.68
Ramachandran favored (%)	97.91	97.84	97.49
Ramachandran allowed (%)	2.09	2.09	2.51
Ramachandran outliers (%)	0.00	0.07	0.00
Rotamer outliers (%)	1.47	1.23	0.99
Clashscore	2.73	2.60	2.66
Average B‐factor (å^2^)	34.89	29.39	25.47
Macromolecules	33.95	27.78	24.52
Solvent	42.69	40.08	32.69
Number of TLS groups			4

*Note*: Statistics for the highest‐resolution shell are shown in parentheses.

*Abbreviations*: TLS, translation/libration/screw.

The structure of d‐methionine bound *N*. *meningitidis* MetQ was solved from 1.68 å resolution diffraction data using a model of liganded MetQ (PDB: 3IR1) with omitted l‐methionine as the search model for molecular replacement in Phenix. The final refinement statistics were *R*_work_/*R*_free_ = 0.17/0.19 (Table 2). The structure of l‐methionine bound *N*. *meningitidis* MetQ was solved from 1.55 å resolution diffraction data using a model of liganded MetQ (PDB: 3IR1) with omitted l‐methionine as the search model for molecular replacement in Phenix. The final refinement statistics were *R*_work_/*R*_free_ = 0.16/0.18 (Table 2).

### 3.6 Isothermal titration calorimetry

Samples of wild type and N238A *N*. *meningitidis* MetQ variants were dialyzed overnight in the purification buffer containing 20 mM HEPES pH 7.5, 150 mM NaCl using a Slide‐a‐lyser mini dialysis device (Thermo Scientific). The samples were then ultracentrifuged at 267,000*g*, 4°C for 20 min to remove aggregates, and the protein concentration adjusted to 50 μM (1.35 mg/mL) and 0.5 mM (13.5 mg/mL) for wild type and N238A MetQ, respectively. d‐ and l‐methionine solutions were prepared in the dialysis buffer (20 mM HEPES pH 7.5 and 150 mM NaCl) of each sample. Titrations of l‐, d‐methionine to MetQ variants were done in triplicate on a MicroCal iTC‐200 calorimeter at 25°C. Titration curves were shown in Figure 1 with detailed protein and ligand concentrations. Data were processed using the software described elsewhere,^25^ with binding constants and enthalpy of associations shown in Table 1.

## AUTHOR CONTRIBUTIONS

P.T.N. and D.C.R. designed experiments, analyzed data, and wrote the manuscript. P.T.N. performed the experiments. J.Y.L. contributed to the design and performance of experiments. J.T.K. contributed to the X‐ray crystallographic data analysis.

## CONFLICT OF INTEREST

The authors declare no conflict of interest.

## Data Availability

Coordinates and structure factors have been deposited in the Protein Data Bank of the Research Collaboratory for Structural Bioinformatics, with PDB IDs 6OJA (l‐methionine‐bound *N*. *meningitidis* MetQ), 6CVA (substrate‐free N238A *N*. *meningitidis* MetQ), and 6DZX (d‐methionine‐bound *N*. *meningitidis* MetQ). The MetQ constructs will be available through Addgene, with IDs 129647 (NmMetQ) and 129648 (N238A NmMetQ).
